# Longitudinal evaluation of the distribution of intraretinal hyper-reflective foci in eyes with intermediate age-related macular degeneration

**DOI:** 10.21203/rs.3.rs-3273570/v1

**Published:** 2023-09-20

**Authors:** Srinivas Sadda, Aditya Verma, Giulia Corradetti, Muneeswar Nittala, Ye He, Marco Nassisi, Swetha Bindu Velaga, Jonathan Haines, Margaret Pericak-Vance, Dwight Stambolian

**Affiliations:** Doheny Eye Institute; University of Louisville; Doheny Eye Institute; Doheny Eye Institute; Institut de la Vision; University of Miami

**Keywords:** Age-related macular degeneration, Intraretinal hyper-reflective foci, Optical coherence tomography, Retinal pigment epithelium

## Abstract

**Purpose:**

Intraretinal hyper-reflective foci (IHRF) are optical coherence tomography (OCT) risk factors for progression of age-related macular degeneration (AMD). In this study we assess the change in the number and distribution of IHRF over two years.

**Methods:**

The axial distribution of IHRF were quantified in eyes with intermediate AMD (iAMD) at baseline and 24 months, using a series of 5 sequential equidistant en face OCT retinal slabs generated between the outer border of the internal limiting membrane (ILM) and the inner border of the retinal pigment epithelium (RPE). Following thresholding and binarization, IHRF were quantified in each retinal slab using ImageJ. The change in IHRF number in each slab between baseline and month 24 was calculated.

**Results:**

Fifty-two eyes showed evidence of IHRF at baseline, and all continued to show evidence of IHRF at 24 months (M24). The total average IHRF count/eye increased significantly from 4.67 ± 0.63 at baseline to 11.62 ± 13.86 at M24 (p<0.001) with a mean increase of 6.94 ± 11.12 (range: − 9 to + 60). Overall, at M24, 76.9% eyes showed an increase in IHRF whereas 15.4% of eyes showed a decrease (4 eyes [7.6%] showed no change). There was a greater number of IHRF and a greater increase in IHRF over M24 in the outer slabs.

**Conclusions:**

IHRF are most common in the outer retinal layers and tend to increase in number over time. The impact of the distribution and frequency of these IHRF on the overall progression of AMD requires further study.

## Introduction

Among the structural optical coherence tomography (OCT) biomarkers in eyes with intermediate age-related macular degeneration (iAMD), intraretinal hyper-reflective foci (IHRF) have emerged as one of the strongest risk factors for progression to late AMD.^1–13^ These IHRF lesions typically correspond to hyperpigmentation on color photographs, which is also a well-established risk factor for AMD progression. Although an inflammatory origin has been proposed,^14^ the majority of reports suggest a dissociation of the dying or stressed retinal pigment epithelial (RPE) cells as a more common source of these lesions.^12,15,16^ Regardless of the origin, both clinic-based and population-based studies have confirmed the prognostic importance of these lesions.^13^

Longitudinal studies suggests that not only the presence, but also an increase in the number of IHRF over time is an independent risk factor for progression to late AMD.^4,8,9,13^ According to Cao et al, these disintegrating cells may get “subducted” underneath the RPE monolayer to enter the sub-RPE space, or get scattered in the intra-retinal compartment.^12, 16–19^ Migration of these cells towards the deep capillary vascular plexus (DCP) is thought to trigger the development of macular neovascularization (MNV).^16,17^ In a recent study, we identified that IHRF present in the outer retina, and in particular the outer nuclear layer, were most strongly associated with the risk for progression to late-stage AMD, though IHRF may be identified in more inner layers as well.^20^ Given the differential risk for progression, we speculated that IHRF present in the more inner retinal layers may differ in their source compared to outer retinal IHRF, but this requires further histopathologic correlation.

While these data highlight that not only the presence, but also the distribution (inner vs outer) of IHRF matters with regards to progression risk, the evolution of these lesions over time is not defined, as most previous studies were cross-sectional nature. This leaves several unanswered questions, for example, does the proportion of IHRF in the inner retina increase in the inner retina over time, suggesting a progressive inward migration of these lesions? Do the numbers of IHRF progressively increase once they appear or are they more dynamic showing a waxing and waning pattern? Understanding the longitudinal evolution of IHRF in intermediate AMD is essential, if quantitative assessment of IHRF is to be used as a biomarker in future studies.

Thus, to address these questions, we conducted a longitudinal analysis of the quantity and distribution (axial location) of IHRF in eyes with intermediate AMD over a 24-month period in a cohort of eyes enrolled in the Amish Eye Study.^10,13^

## Methods

The details of the Amish Eye Study (supported by NEI R01EY023164 and 1R01EY030614) have been described in prior reports. Briefly, the Amish Eye Study was a longitudinal prospective observational study aimed at understanding the OCT-based risk factors and their genetic association with AMD progression.^10^ All subjects in the study (N = 1339) underwent baseline volume OCT assessment and approximately half of the individuals (N = 666) returned for a 24-month follow-up visit including OCT imaging. All participants in this IRB-approved study signed written informed consent. The study was performed in accordance with the Health Insurance Portability and Accountability Act and adhered to the tenets of the Declaration of Helsinki.

OCT volume scans were obtained of both eyes of all subjects using the Cirrus OCT (Carl Zeiss Meditec, Dublin, CA; 512×128 macular cube; 6×6 mm scan region centered at the fovea). Deidentified OCT volumes were exported and transmitted to the Doheny Image Reading and Research Lab for analysis. To be eligible for this analysis, subjects had to have evidence of intermediate AMD (Beckman classification)^21^ and IHRF on the baseline OCT scans. Eyes with evidence of a retinal disease other than iAMD (evidence of early or late AMD, diabetic retinopathy, epiretinal membrane, etc) were excluded. Eyes with OCT scans with gross segmentation errors which could impact retinal slab selection and poor-quality images were also excluded.

## OCT Analysis Protocol:

**Figure 1:**
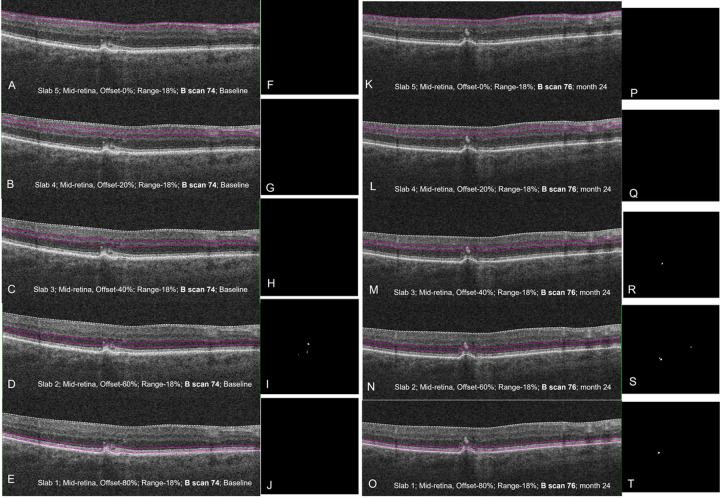
Structural OCT B scan with corresponding slab locations at baseline (A, slab 5 to E, slab 1), and month 24 (K, slab 5 to O, slab 1), showing the presence of intra-retinal hyperreflective foci, with corresponding depiction of these lesions on the en-face images extracted using imageJ. Note the presence of IHRF lesions in slab 2 (Fig 1D and 1I) at baseline, which were similar in number but at different locations at Month 24 (Fig 1D and 1I). Note, new IHRF appeared in Slab 1 (Fig 1O and 1T) and slab 3 (Fig 1M and 1R).

Assessment of the quantity and distribution of IHRF across various levels of the retina requires assessment of multiple en face OCT slabs through the mid-retina. The methodology for generating these slabs and quantitatively analyzing IHRF has been detailed in prior reports.^20,22,23^ In brief, the procedure includes the following steps: 1) identification of the presence of IHRF on structural OCT B-scans (identified as well-circumscribed hyperreflective lesions ≥ 3 pixels in size, located within the neurosensory retina); 2) generation of 5 equidistant en-face slabs generated from the mid-retina (each representing 20% of the retinal thickness between the RPE and ILM with the inner surface of the slab following the ILM contour and the outer surface following the RPE contour, using the offset and range function); 3) thresholding and binarization of slabs with ImageJ (version 1.50; National Institutes of Health, Bethesda, MD; available at http://rsb.info.nih.gov/ij/index.html);^24^ 4) manual removal of other hyper-reflective artifacts surrounding the IHRF (drusen, subretinal drusenoid deposits, blood vessels etc); and finally, 5) automatic quantification (using ‘analyze particles’ function in Image J) of IHRF (number) from each retinal slab thus generated. This procedure was followed for each eye at baseline and at the 24-month follow up (Fig. 1). The slabs were numerically ordered with slab 1 being the outermost (closest to the RPE layer- the presumed source of many of these lesions) and slab 5 being innermost.

## Statistical analysis:

Statistical analyses were performed using SPSS Statistics version 21 (IBM, Armonk, NY). Qualitative features were described by frequency (n) and percentage (%). Descriptive statistics were reported as means ± standard deviations. The 24-month change in the number of iHRFs in the total retina and each individual slab was assessed using paired t-tests. A P < 0.05 was considered statistically significant.

## Results

Of the 666 subjects with two years of follow-up data in the Amish Eye Study, 120 eyes of 71 subjects had evidence of iAMD at baseline. The mean age ± standard deviation [SD] of these subjects was 72.27 ± 10.52 years, 36 (50.7%) were females. Of these 120 eyes, 52 eyes (43.3%) from 42 subjects had evidence of both iAMD and IHRF, and this group constituted the final analysis cohort for this study [mean age ± SD = 74.17 ± 9.37 years; 24 (57.1%) females]. All eyes continued to show the evidence of IHRF at the 24-month follow-up visit. IHRF were seen in both eyes in 10 subjects (20 eyes), and in only one in 32 subjects (16 each in the right and the left eye).

**Figure 2:**
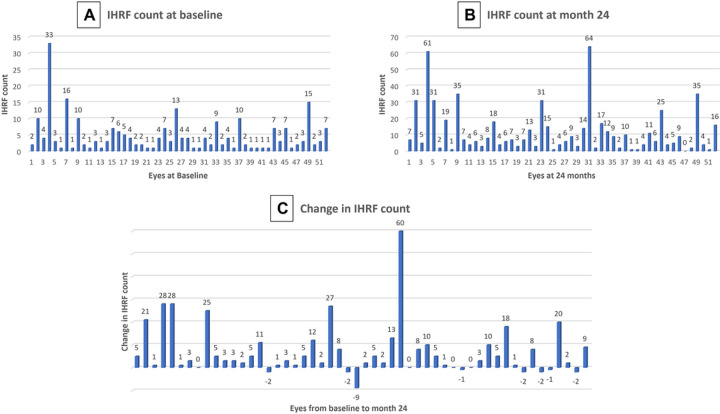
Frequency histograms showing the number of IHRF in all 5 en face slabs combined for each case at baseline (A) and Month 24 (B), and the difference (C) between Month 24 and baseline.

Quantitative analysis revealed that the total IHRF count/eye (entire retina – all 5 slabs) increased significantly from 4.67 ± 5.39 (mean/eye± standard deviation, SD; range = 1 – 33; median = 3) at baseline to 11.61 ± 13.72 (range = 0–64; median = 6.5) at M24, and this difference was statistically significant (p < 0. 001). The average change over the 24 months was + 6.94 ± 11.12 (range = − 9 to + 60; median = + 3). Figure 2 illustrates the IHRF counts at baseline, M24, and the change over two years for all eyes in the study cohort. At month 24, an overall reduction in the IHRF count was noted in 8 of the 52 eyes (15.4%), 4 eyes (7.6%) showed no change, and the IHRF count increased in the remaining 40 eyes (76.9%).

**Figure 3:**
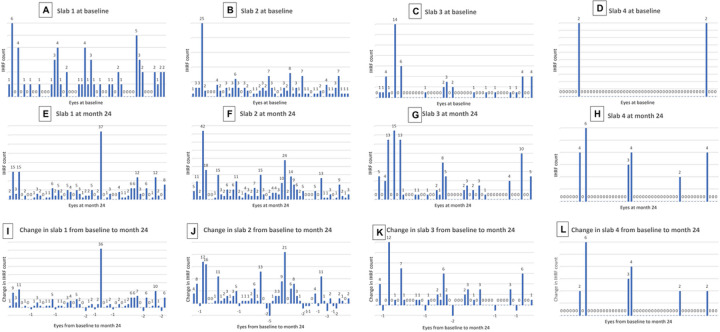
Frequency histograms showing the number of IHRF in individual en face slabs (1–4) for each case at baseline (A-D) and Month 24 (E-H), and the difference (I-L) between Month 24 and baseline. Slab 1 (A, E, I) is the outermost slab, closest to the retinal pigment epithelium. The positions of the slabs are shown in Figure 1.

With regards to the distribution of IHRF for the entire cohort, at baseline 24.27% were located in slab 1, 54.32% in slab 2, 19.75% in slab 3, 0.016% in slab 4, and 0% in slab 5 (the innermost slab). At 24 months follow-up, 32.11% were located in slab 1, 47.51% in slab 2, 16.55% in slab 3 and 0.038% in slab 4, whereas slab 5 continued to show no IHRF lesions. The IHRF counts in the individual slabs at baseline, M24, and the 24 months change for Slabs 1–4 is shown in Fig. 3. Overall, 17 of the 52 eyes (32.7%) showed a decrease in the IHRF count in at least one slab. The percentage of cases showing a decrease, increase, or stable IHRF count in each slab is shown in Table 1. Overall, it is apparent that the maximum increase is noted in the outer retinal slabs (spanning from inner border of RPE till the outer nuclear layer, ONL) with a small percent of eyes showing a decrease, whereas the inner slabs showed a stable count in the majority of cases.

## Discussion

In this study, we demonstrated that once IHRF are noted to be present, they can increase dramatically over 24 months (2.5x fold on average). In addition, once present, they persist over at least 24 months, though a minority (~ 15%) of eyes can demonstrate a decrease in the number of IHRF over this timespan. While the overall trend is for IHRF to increase, at a given location the presence of IHRF can change dynamically with lesions appearing or disappearing. The dramatic increase in IHRF counts over time is most evident in the outermost retinal layers, whereas the IHRF counts overall tend to be stable in the inner retinal layers.

Indeed, as shown in Table 1, the IHRF counts in the outer slabs (1 and 2) increased in ~ 2/3 of cases, whereas it only increased in 11.5% of cases slab 4, and IHRF never appeared in the innermost slab 5 throughout the 24-month period of study. These results would appear to argue against a continuous and progressive inward migration of IHRF as has been reported previously.^4,12^ IHRF have been suggested to originate from either the degenerating RPE or from microglia.^12,14–16, 25,26^ The inward migration of distressed RPE cells towards DCP has been implicated as a precursor and precipitating factor for the development of type 3 MNV.^16,17^ Our results do suggest that there is a dramatic increase in IHRF over time in the outer retina up to the DCP. This rate of increase, however, does not appear to continue beyond the DCP level. To explain this observation, we hypothesize that the intraretinal migration of RPE cells is triggered by underlying choriocapillaris ischemia and the RPE cells are drawn inward by the possibility of perfusion and nourishment from the retinal DCP. Thus, once reaching the DCP vasculature, there is no further ischemic drive or oxygenation gradient to pull the RPE cells further inward. If one were to accept this explanation, how can one account for the presence of some IHRF lesions (albeit at lower frequency) at some inner retinal levels. We would theorize that many of these IHRF in the inner retina layers may be of a different source, possibly of inflammatory or microglial origin.^27^

Further supporting the hypothesis of a differential origin of inner and outer retinal IHRF, in a recent analysis (unpublished) we observed that the risk of progression to late AMD was primarily attributable to outer retinal IHRF. This would be consistent with the concept that outer retinal IHRF are primarily of RPE origin and a reflection of choriocapillaris ischemia. While these hypotheses require histopathologic confirmation, the possibility of multiple sources of IHRF highlight that multiple pathophysiologic pathways may be at play during the progression the AMD, and this has potential implications for future therapeutic development.

In addition, to the changes in distribution over time, another important observation of this longitudinal analysis is the dramatic increase in IHRF count over the 24-month period. This suggests that the appearance on IHRF may represent a critical tipping point in the eye, highlighting an eye that may be in severe distress. Once an IHRF appears, there may be dramatic disease acceleration with many more IHRF appearing. This is not surprising, as IHRD are perhaps the strongest biomarker for progression to late AMD and atrophy.^1,4–9,13^ This might also highlight a potential intervention point for future therapeutics. Pegcetocoplan has recently been cleared by the FDA for treatment of geographic atrophy, and approval of avacincaptad is anticipated in the near future.^28–30^ Post-hoc analyses suggest that these agents may be even more effective if given earlier in the disease process, reducing conversion from incomplete RPE and outer retinal atrophy (iRORA) to complete RORA (cRORA).^31^ Earlier intervention at the first appearance of IHRF may be a target for future therapies and trials, and prevention of an increase in IHRF could potentially serve as an endpoint to assess effectiveness of treatment.

This study is not without limitations. The number of eyes with both iAMD and IHRF were relatively small, and we only had 2 years of follow-up data. Second, the Amish are a relatively homogenous population with regards to both environmental (such as no or minimal smoking, similar diet etc) and genetic factors, and therefore, the results may not be generalized to other population groups.^10^ Third, we quantified the distribution of IHRF lesions, but did not quantify their area or other characteristics such as their reflectivity or shape. Fourth, while we assessed the position of IHRF relative to the ILM and RPE, we did not quantify IHRF within specific retinal layers. On the other hand, segmentation of retinal layers in eyes with AMD can be challenging and may not be reliable, particularly over drusen where the outer retinal bands are commonly not well seen because of the altered reflectivity. Finally, while we quantified overall numbers of IHRF within an eye, we did not systematically track progression or regression of individual IHRF lesions. This can be a topic of future studies, particularly with datasets including more frequently obtained OCT scans.

Our study also has several strengths including the use of a standardized OCT acquisition protocol, dense volume OCT scanning, and the use of certified reading center OCT graders with a previously demonstrated high level of reproducibility.

In summary, IHRF can dramatically increase in number over time, particularly in the outer retina. The increase in IHRF in the inner retina is less extensive, suggesting that IHRF originating from the RPE tend to not migrate beyond the outer retina. This suggests that IHRF in the inner and outer retina may have a different origin and pathophysiology. Regardless, IHRF counts over time may be a quantitative measure of disease progression and may serve as biomarkers or endpoints in future therapeutic trials. Further longitudinal studies, however, are required.

## Figures and Tables

**Table 1:** The percentage of cases showing a decrease, increase, or stable IHRF count in each slab.

Retinal slabs	Increase (%)	Decrease (%)	No change (%)
Slab 1 (outermost)	65.5	13.4	21.1
Slab 2	67.4	21.1	11.5
Slab 3	36.6	9.6	53.8
Slab 4	11.5	0	88.5

## Summary

### What was known before:

1. Intraretinal hyper-reflective foci are optical coherence tomography biomarkers that are associated with an increased risk for progression from intermediate age-related macular degeneration to late AMD.
2. These lesions are believed to correspond to either degenerating retinal pigment epithelium or inflammatory microglia.

### What this study adds:

1. IHRF are found mainly in the outer retinal layers and once present in an eye, their numbers tend to increase over time in the vast majority of cases.
2. The maximum increase in IHRF numbers is observed in the outer retinal layers, whereas the number of IHRF in the inner retinal layers tends to remain stable. This may point to a different pathogenesis or origin of IHRF in the different layers.
